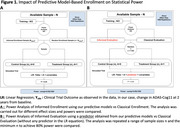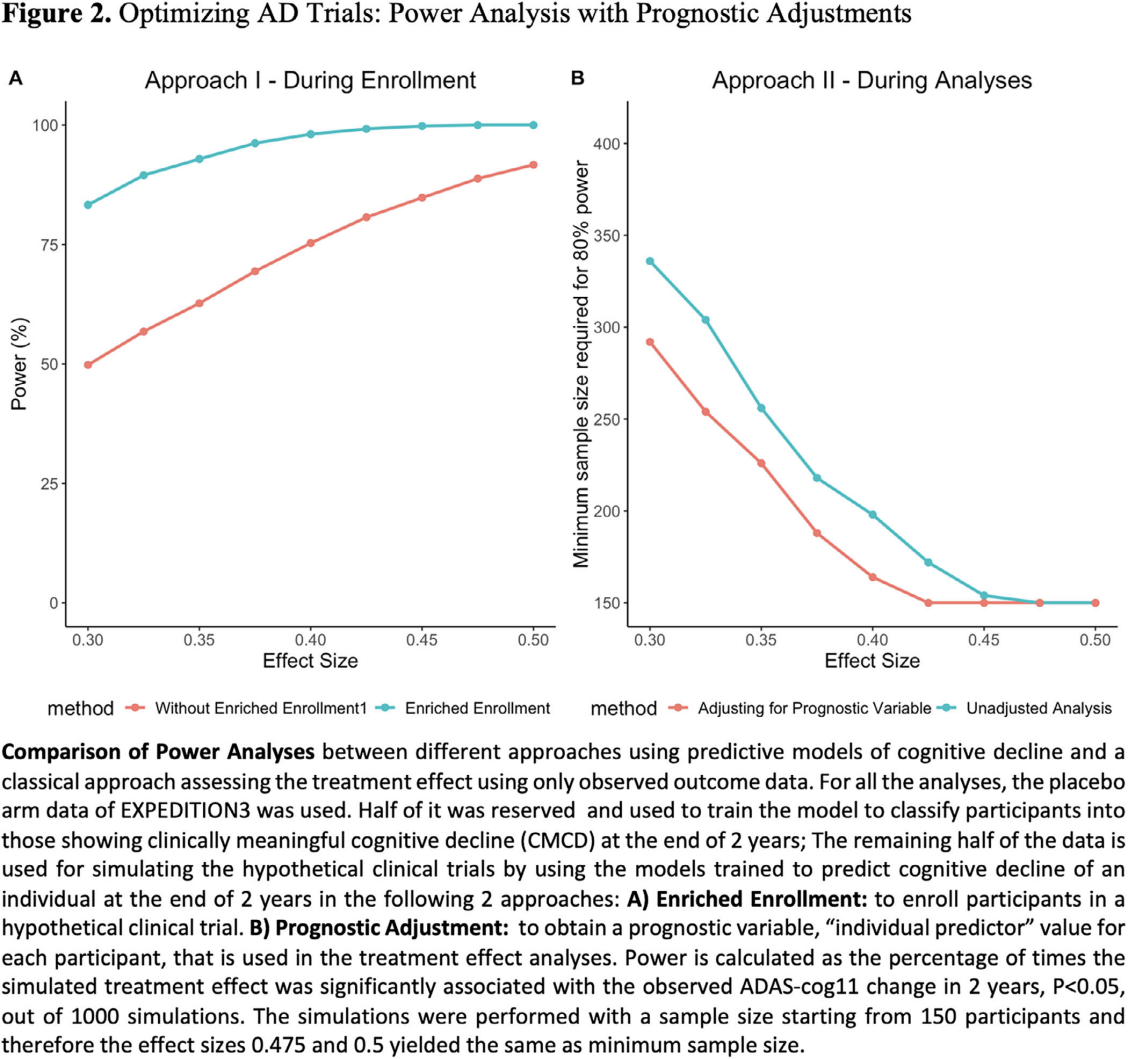# Optimizing Alzheimer’s Disease Clinical Trials with Predictive Models of Cognitive Decline

**DOI:** 10.1002/alz70859_098567

**Published:** 2025-12-25

**Authors:** Bhargav Teja Nallapu, Tianchen Qian, Richard B. Lipton, Ali Ezzati

**Affiliations:** ^1^ Technical University of Delft, Delft, Zuid‐Holland Netherlands; ^2^ Albert Einstein College of Medicine, Bronx, NY USA; ^3^ University of California, Irvine, Irvine, CA USA; ^4^ Department of Neurology, Albert Einstein College of Medicine, Bronx, NY USA

## Abstract

**Background:**

In Alzheimer’s Disease (AD) treatment trials, up to 40% of placebo arm participants do not show cognitive decline over the course of treatment, reducing power to detect treatment effects. Enrolling persons predicted to have cognitive decline if treated with placebo could support enriched enrollment potentially increasing power to detect treatment effects.

**Method:**

Using data from 1072 patients in the EXPEDITION3 trial (placebo arm), we developed machine learning classifiers to predict Clinically Meaningful Cognitive Decline (CMCD, change in ADAS‐Cog≥3) ) by the end of the trial (week 80). The classifiers used demographics, neuropsychological tests (NP) and biomarkers, including APOE4 genotype and volumetric MRI as features. The classifiers were trained on 50% of the data, while the remaining 50% was used for plasmode simulations of hypothetical clinical trials. In each of the simulations, a classical analysis of treatment effects was performed using linear regression with change in ADAS‐cog11 at the end of the trial as the outcome and treatment indicator as a covariate, assuming the same treatment effect on every participant. The plasmode simulations were performed in two separate approaches: (1) Enriched Enrollment, only enrolling participants predicted to experience CMCD if treated with placebo, and (2) Prognostic Adjustment, incorporating a predicted decline variable into treatment effect analyses. (Figure 1).

**Result:**

Participants from the placebo arm of the EXPEDITION3 trial were on average 72.7±7.7 years old, and 59% were female. CMCD occurred in 55.8% of placebo‐treated participants by the end of the trial. For treatment effect sizes of 0.3 to 0.5, enriched enrollment consistently achieved higher power than standard enrollment (same sample size as enriched enrollment, Figure 2A). Prognostic adjustment reduced the minimum sample size required for 80% power (e.g., for effect size = 0.3, adjusted analysis required 292 participants vs. 336 for unadjusted analysis) (Figure 2B). The benefit of both approaches diminished as effect sizes increased but remained superior to standard methods.

**Conclusion:**

Our results demonstrate that predictive models have the potential to optimize AD trial design by selecting participants expected to decline if treated with placebo and refining the analyses of treatment effects with prognostic adjustments.